# PRO-Act: a healthcare provider workshop outlining the added value of implementing PROs in routine HIV practice

**DOI:** 10.1186/s41687-023-00584-w

**Published:** 2023-06-01

**Authors:** António Antunes, Ricardo Racha-Pacheco, Catarina Esteves, Ana Tavares, Josefina Mendez, Patrícia Pacheco, Duncan Short

**Affiliations:** 1 ViiV Healthcare Medical Department, Rua Dr. António Loureiro Borges, nº 3 - Arquiparque - Miraflores, 1495-131 Algés, Portugal; 2 Hospital CUF Descobertas, Rua Mário Botas, 1998-018 Lisbon, Portugal; 3 Infectious Diseases Ward, Hospital de Cascais, Av. Brigadeiro Victor Novais Gonçalves, 2755-009 Alcabideche, Portugal; 4 Hospital Santo António, Largo Do Prof. Abel Salazar, 4099-001 Porto, Portugal; 5 Hospital Fernando da Fonseca, IC19, 2720-276 Amadora, Portugal; 6 ViiV Healthcare Implementation Science Department, 980 Great West Road, Brentford Middlesex, TW8 9GS UK

## Abstract

Patient Reported Outcomes (PROs) are an evidenced way of adding value to routine clinical care. As a source of unique information on the effect of a medical condition and its treatment from the patients’ perspective (Mercieca-Bebber et al. in Patient Relat Outcome Meas 9: 353–367, https://doi.org/10.2147/PROM.S156279, 2018), PROs allow for an improved assessment in routine clinical care of symptoms, side effects, functional outcomes (physical, sexual, social, emotional, cognitive functioning), and health-related quality of life (HRQoL). By helping to align healthcare providers’ interventions with what matters most to the patient, PROs contribute to the individualized choice of Anti-Retroviral Therapy (Carfora et al. in PLoS ONE 17(4): e0267030, https://doi.org/10.1371/journal.pone.0267030, 2022) as a paramount component of tailored and person-centred care management, in routine clinical practice. This article outlines a practical framework and process tested in Portugal to raise awareness of PROs’ added value, and to help guide first steps in the implementation and seamless integration of PROs, in routine HIV care.

**Supplementary Information:**

The online version contains supplementary material available at 10.1186/s41687-023-00584-w.

## PROs in modern HIV care

The advent of antiretroviral therapy (ART) has led to profound changes in the clinical outcomes for people living with HIV (PLHIV), resulting in a reduction of HIV associated mortality, improved prognosis, and increased life expectancy—transforming this disease into a manageable, chronic condition [1, 2]. However, managing the aging phenomenon in PLHIV presents challenges, such as increased co-morbidities [3] and a reduced health-related quality of life (HRQoL) [4] when compared with the general population.

PLHIV have multidimensional concerns affecting HRQoL, including issues such as stigma, discrimination, psychosocial issues arising from aging with HIV, communication and shared decision-making with their healthcare professionals (HCPs) [5]. Thus, in addition to the 90-90-90 goals established by UNAIDS (the Joint United Nations Programme on HIV and AIDS), which aim to end the AIDS epidemic by 2030, by improving diagnosis, treatment, and viral suppression in PLHIV [6], experts have advocated for an equally important “4th 90”: Ensuring that 90% of PLHIV with viral suppression have good HRQoL [7]. Subsequent to this objective, the focus and paradigm of HIV care has evolved, from managing disease-associated morbidity and mortality to addressing issues such as symptom burden and HRQoL. Modern HIV care needs to evolve to tackle the complex personal challenges of each individual and become more integrative and person-centred, responsive to physical, therapeutical, social, psychological, cognitive, and informational needs of PLHIV [8]. This holistic and individualized approach to HIV care will ultimately allow PLHIV to be better understood, receive more relevant support and be more involved in their treatment; thus, leading to better health outcomes [9].

One of the most effective ways to help healthcare providers understand the needs and priorities of patients is the collection of patient-reported outcomes (PROs). PROs have been defined by the US Food and Drug Administration (FDA) as “any report of the status of a patient’s health condition that comes directly from the patient, without interpretation of the patient’s response by a clinician or anyone else” [10].

The gathering of PROs before the patient and provider meet allows for the assessment of several dimensions of care relevant to a patient visit: physical symptoms, mental health, functioning abilities, life circumstances, social well-being and HRQoL, among others [11, 12]. These aspects can correspond to individual’s perceptions regarding own health which could not be measured otherwise (e.g., pain, fatigue) or that are not easily observable (e.g., adherence to therapy, mental health, substance use, risk behaviours) [13]. PROs stand as complementary to biological measures and physical examinations in routine clinical care—both subjective and objective views of health being considered; allowing HCPs to evaluate the health status of each patient and how the individual feels about the care provided [14]. Thus, collecting PROs allows for the identification of patients’ needs and priorities that may otherwise not be identified by HCPs in time-limited appointments, enabling the HCP to effectively direct and focus healthcare interactions and care [11]. PROs are an opportunity to understand a person and their health, in a multidimensional and holistic way, beyond clinical data.

In HIV care, personalised care and the application of PROs is particularly relevant, as many difficult to detect issues—including substance use, depression, violence in intimate relationships—are more prevalent than in the general population [11, 15], and many symptoms, health, sexual risk behaviours, and life circumstances of PLHIV are not directly observable—thus being frequently overlooked by HCPs. Subjective aspects evaluated through PROs, such as pain, general health perception, and life satisfaction have also been demonstrated as valid clinical predictors of hospitalization and mortality [13, 16].

The application of PROs for routine clinical care is not well established in HIV care. PROs have been primarily used in clinical trials, relating to the therapeutic outcomes [17]. More recently PROs use in clinical practice has been studied and implemented in a broader practice of HIV care, having the potential to improve three fundamental areas: supporting HCPs in providing the best patient-centred care; measuring and comparing the quality of care; providing data to evaluate health practices and policies [18]. Specifically, the use of PROs in the clinical context of HIV care has been shown to: improve the communication between HCPs and patients, effectively structure clinical appointments and optimize time management [19–23]; allow for the identification of behaviours and/or conditions otherwise difficult to observe, such as depression, anxiety [21], substance use, and inadequate adherence to ART [19, 21, 24, 25]; and improve PLHIV self-knowledge, empowering them as partners in their own care, ultimately resulting in improved outcomes in the management of HIV infection [26, 27]. Additionally, PLHIV have found PRO collection prior to their appointments to be acceptable, reliable, and easy to perform [20–22, 28].

Using PROs to record PLHIV experiences with ART (i.e., patient-reported symptoms) was proven to be a more accurate way to predict clinical outcomes than exclusively using adverse effects reported by HCPs [13, 29, 30]. Simultaneously, PROs have been shown to be feasible to implement in routine care with minimal disruption upon clinic flow and acceptable to patients and providers as part of this process [21].

## Building the PRO-Act workshop

Notwithstanding their proven clinical relevance, PROs are not yet routinely integrated in Portuguese HIV care, mainly due to the uncertainty on how to implement and customize them to each site-specific reality, despite available guidance [13, 18, 31]. Most of these limitations may be overcome through structured implementation, hence the need for a workshop to enhance awareness on the available resources.

**Fig. 1:**
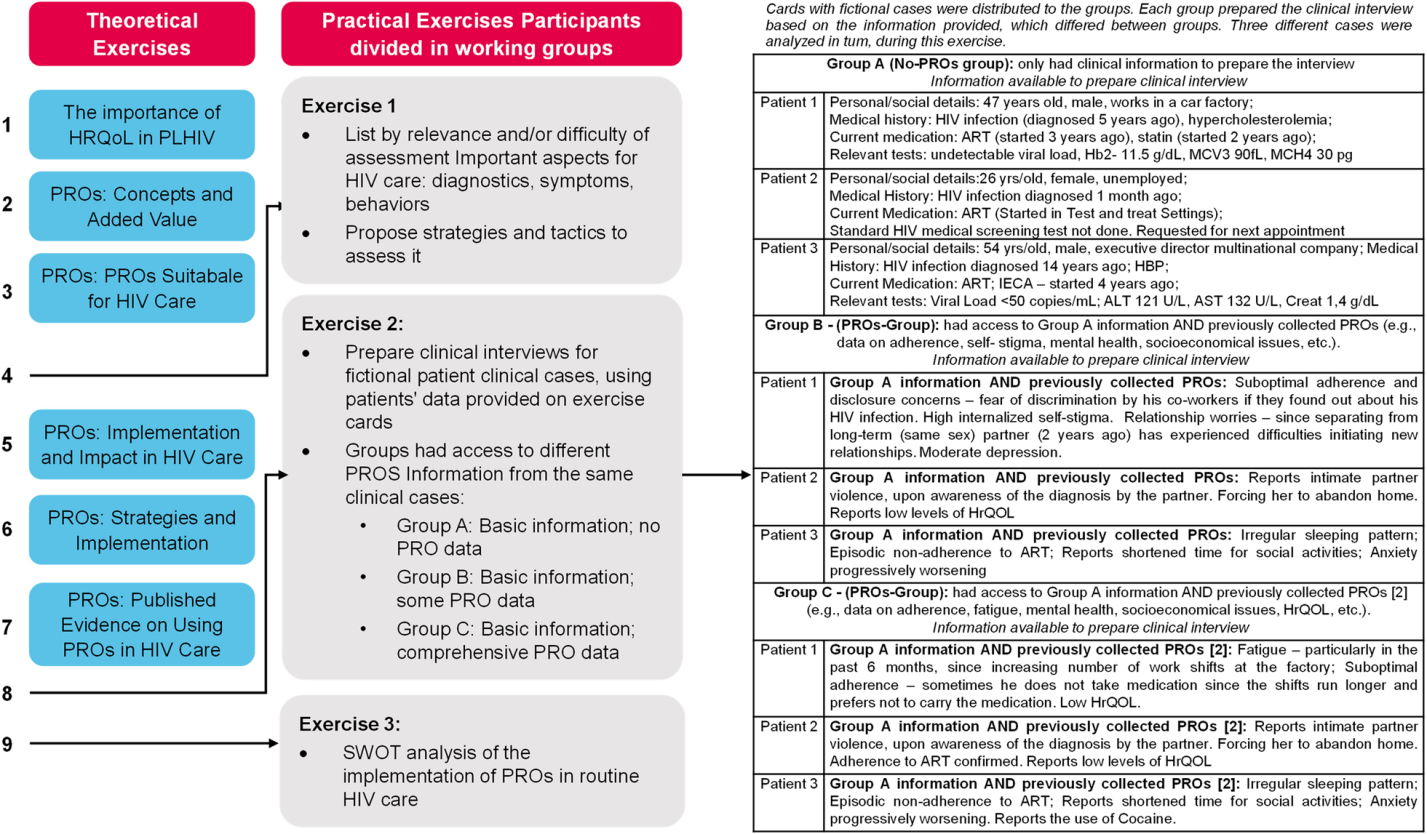
PRO-Act Workshop agenda: the workshop consisted of six brief theoretical presentations combined with three interactive practical exercises. During the latter, participants were incited to participate in the discussion by the workshop’s facilitator

The «PRO-Act» project aims to raise awareness on the added value of PROs integration in routine HIV care and available implementation resources, and to provide a framework boosting its uptake in Portuguese HIV care. The first activity was a day-long master-workshop, in which Portuguese HCPs (physicians, nurses, pharmacists) were informed, by national and international speakers, on the relevance of PROs, available resources, and use in routine clinical HIV care. To this end, the PRO-Act workshop included six theoretical components (HRQoL in PLHIV; PROs: concepts and added value; PROs: new tools; PROs: framework and impact; PROs: strategies and implementation; and PROs: published evidence, Fig. 1), which provided an evidence-based context on the different aspects of PROs in HIV care and promoted interactive sessions where the experts shared their experiences with the participants. However, the most innovative aspect of the workshop was the strong practical component (Fig. 1). In the practical sessions the participants were asked to: reflect on diagnostics, symptoms, and behaviours which evaluation is important or difficult in HIV care, and propose strategies to tackle them; prepare clinical interviews for fictional clinical cases, using patients’ data provided on exercise cards, where some groups had access to PROs' information and others did not; and perform a SWOT analysis, evaluating the strengths, weaknesses, opportunities, and threats of the implementation of PROs in routine HIV care (Fig. 1).

## Outputs and feedback from the PRO-Act workshop

Twenty Portuguese HCPs—Physicians, Nurses and Pharmacists with different levels of expertise (from National Key Opinion Leaders to young specialists), caring for 100 to 800 PLHIV per HCP, and working in the largest centres in Portuguese HIV care—were directly invited and participated in the first PRO-Act master-workshop—Lisbon, March 2022.

The main and innovative focus of the workshop were the practical exercises in which the participants were divided into three work groups.

### First exercise

In this introductory exercise the groups listed the aspects of HIV care they considered to have the greatest relevance in clinical practice (Table 1). The outputs from the groups were: neuropsychiatric symptoms, socioeconomic conditions, patient-HCP communication (group 1); depression, alcohol consumption, self-stigma (group 2); neurocognitive changes (memory, sleep quality), sexuality (maternity, breastfeeding, contraception, relationships), substance abuse, adherence to therapy (group 3).

**Table 1:** Aspects of HIV care, considered to have the greatest relevance in clinical practice, as listed by the groups

Exercise 1		
Aspects of HIV		
Considered of greatest relevance in **Clinical** Practice, by the participants	Deemed as most difficult to assess **in HIV** Clinical Practice, by the participants	Potential Strategies to Overcome Assessment Challenges in Clinical Practice, proposed by the participants
Neuropsychiatric symptoms, Socioeconomic conditions, Patient—HCP communication Depression, Alcohol consumption, Self-stigma_,_ Neurocognitive changes (memory sleep quality), Sexuality (maternity, breastfeeding_:_ contraception, relationships), Substance abuse, Adherence to therapy	Neuropsychiatry symptoms, Sexual risk behaviour. Addictive behaviours (alcohol, drugs), Changes in memory, Substance abuse [drugs), Stigma, Adherence to therapy, Domestic/work violence, discrimination	Anonymous questionnaires. Strategy adaptation to individual cultural context and literacy level. M u Itid isci plina ry teams, Promotion of simultaneous direct contact with HCPs from different medical specialties. Collaboration with community based non-governmental organizations (NGOs). Communication improvement between patients, physicians, nurses, NGOs, and healthcare administrators. Improvement of patients and general population (e.g., in schools) health literacy. Better communication skills of HCPs: focusing on sensibility and empathy. Training the HCPs to correctly interpret and to act accordingly to PROs' findings

The groups also presented the aspects of HIV care that they deemed as most difficult to assess in HIV clinical practice, encompassing: neuropsychiatric symptoms, sexual experiences, addictive behaviours (alcohol, drugs) (group 1); changes in memory, substance use (drugs), stigma (group 2); adherence to therapy, domestic/work violence, discrimination (group 3).

Noticeably, several of the aspects of greatest relevance overlapped with the aspects considered as having greatest assessment challenges, emphasizing the requirement for improved assessment strategies. As potential strategies, the participants suggested: anonymous questionnaires; strategy adaptation to individual cultural context and literacy level; multidisciplinary teams, promoting simultaneous direct contact with HCPs from different medical specialties; collaboration with community-based non-governmental organizations (NGOs); improving communication between patients, physicians, nurses, NGOs, and healthcare administrators; increase patients’ and general population’s (e.g., in schools) health literacy; better communication skills of HCPs: focusing on sensibility and empathy; and training HCPs to correctly interpret and act accordingly to PROs’ findings. Overall, the approaches suggested highlighted the importance of the structured collection of each individual patient’s experiences, enabling the provision of holistic and individualized care—a strategy in which PROs play a key role.

### Second exercise

In this main exercise of the workshop, cards with fictional clinical cases were distributed to the groups. Each group had to prepare clinical interviews suitable to the information provided, for 3 clinical cases. However, the groups had access to different information from the same clinical case: Group A (No-PROs-group) had only clinical information to prepare the interview (e.g., age, gender, occupation, therapies, lab results); Group B (some PRO data group) had previously collected PROs data (e.g., data on adherence, substance abuse, mental health, socioeconomical issues, etc.) in addition to Group A data; Group C (comprehensive PRO data group) had all Group B data, plus more comprehensive PRO data. The objective of presenting the groups with different information pertained to highlighting the importance of PROs in efficiently identifying important issues. The effectiveness of each group was measured by how many of the topics for the clinical interview identified by each group corresponded to the patient-specific issues.

The clinical cases were discussed in turn among all participants. The information available to prepare one clinical interview is shown in Table 2 and the topics the respective groups chose to address during the consultation are presented in Table 3.

**Table 2:** Clinical case #1 [Patient 1]—Information available to prepare the clinical interview. Cards with fictional cases were distributed to the groups. Each group prepared the clinical interview based on the information provided, which differed between groups

Group	Information available to prepare the clinical interview
Group A	Personal/social details: 47 years old, male, works in a car factory Medical history: HIV infection (diagnosed 5 years ago), hypercholesterolemia Current medication: ART^1^ (started 3 years ago), statin (started 2 years ago) Relevant tests: undetectable viral load, Hb^2^ 11.5 g/dL, MCV^3^ 90 fL, MCH^4^ 30 pg
Group B	Group A information AND previously collected PROs [2]: Suboptimal adherence and disclosure concerns—fear of discrimination by his co-workers if they found out about his HIV infection; High internalised stigma; Relationship worries—since separating from long-term (same sex) partner (2 years ago) has experienced difficulties initiating new relationships; Moderate depression
Group C	Group A information AND previously collected PROs [1]: Fatigue—particularly in the past 6 months, since increasing number of work shifts at the factory; Suboptimal adherence—sometimes he does not take medication since the shifts run longer and prefers not to carry the medication; Low health-related quality of life (HRQoL)

^1^ART–antiretroviral therapy; ^2^Hb—hemoglobin; ^3^MCV—mean corpuscular volume: ^4^MCH—mean corpuscular hemoglobin

In general, the groups effectively identified several relevant issues for clinical practice. The PROs-groups managed to successfully address more relevant and patient-specific issues, conducting the clinical interviews more efficiently than the No-PRO-group. In this exercise, the participants experienced first-hand the importance of collecting PROs to structure clinical appointments prioritizing the specific and personal issues of PLHIV. Often, the No-PROs-group was unable to identify important issues that are difficult to observe, for example, the need for social support, and the presence of depression in clinical case #1 (Table 3).

**Table 3:** Clinical case #1 [Patient 1] – Topics working groups chose to address in clinical interviews. Group A (no-PROs-group) conducted the first clinical interview, followed by groups B and C (PROs-groups), which conducted the interviews suggesting their own topics (presented below when topics were added or adjusted by subsequent work groups)

Group	Topics for the clinical interview	
Group A	General symptoms Fatigue and diet Gastrointestinal health	Lifestyle and sleeping habits Changes to usual medication Working in shifts and professional life
Group B	Medication adherence and tolerance Situation at work, changes in schedule Medication at work Depression (evaluation scale)	Relationship with family Social support systems Weight loss Changes in diet
Group C	Fatigue, shifts at work Expectations for ART therapy Relationships at work Workplace support systems	Reinforce U = U—"Undetectable equals Untransmittable” Offer HIV testing for partner Alcohol consumption

### Exercise 2

The PRO groups effectively identified several issues relevant to clinical practice.

The no-PRO-group was less able to identify important issues that are difficult to observe, for example, the need for social support and the presence of depression in case #1.

### Third exercise

In the final exercise, the closing summary, participants did a SWOT analysis of the implementation of PROs in routine HIV care in Portugal, highlighting its strengths, weaknesses, opportunities, and threats—Table 4. The strengths perceived by the participants were: improved communication between patients and HCPs, enhanced problem identification, time optimization, creation of interdisciplinary channels, increased HIV care team motivation, improved patient satisfaction, and autonomy to develop local PROs’ pilot projects. Challenges identified were the difficulty in connecting already existing patient information to new PROs data, and logistical issues for the patients to fill out the questionnaires (i.e., how to get the information to the HCPs, who should oversee and explain).

Participants recognized as opportunities: the fact that even the smallest action would add value (compared to the absolute lack of PROs); the useful availability of this new kind of data for consciousness raising activities. The threats pointed out by the participants were: lack of quick and useful reactions (i.e., concrete actions by the HCPs) considering the pre-workshop situation, and lack of community-based organizations’ responses.

**Table 4:** Results from the SWOT analysis on the implementation of PROs in routine HIV care in Portugal.

Exercise 3	
Participants’ analysis of the implementation of PROs in routine HIV care in Portugal	
Strengths	Challenges
Improved communication between patients and HCPs; Enhanced problem identification; Time optimization; Creation of interdisciplinary channels; Increased HIV care team motivation; Improved patient satisfaction; Autonomy to develop local PROs' pilot projects	Difficulty in connecting already existing patient information to new PROs data; Logistical issues for the patients to fill out the questionnaires (i.e., who would oversee and explain, how to get the information to the HCPs)
Opportunities	Threats
Even the smallest action would add value (comparing to the absolute lack of PROs); Useful availability of this new kind of data for lobbying activities	Lack of quick and useful reactions (i.e., concrete actions by the HCPs) considering the pre workshop situation; Lack of community-based organizations responses

### Feedback

At the end of the workshop, feedback was collected from twenty participants via a short semi-structured questionnaire (Additional file 1: Table S1). The workshop contents were considered pertinent or very pertinent by 100% of the audience, and 90% considered the information shared on the use of PROs to be impactful or very impactful in their clinical practice. Perceived strengths were the use of high-quality practical exercises, the structure, build and dynamics of the workshop, the quality of the theoretical contextualization and presentations, and the workshop’s communication of the potential real-world impact on their clinical practice of PROs. Eighteen (i.e., 90%) participants considered the implementation of PROs in HIV care in Portugal as feasible. On recommendations for future editions of the workshop, several participants suggested the inclusion of more operational details concerning specific PRO scales and a supplementary exercise: seeing, understanding and practicing the use of scales/tools in real-life mock situations (i.e., Using actors as PLHIV).

## Conclusion

Through theoretical and practical sessions, the PRO-Act’s master-workshop provided an overview of PROs and its added value in HIV care. Particularly, by the use of practical exercises using PLHIV examples in both the presence and absence of previously collected PROs, the participants could fully grasp its impact in successfully addressing relevant patient-specific issues, conducting clinical interviews with more efficient approaches. Our methods and strategy demonstrated to HCPs how PROs go beyond clinical data, helping to fully and multidimensionally understand and act on PLHIV’s needs, resulting in better health outcomes and better HRQoL in HIV Care. The feedback collected after the workshop will inform future initiatives of the «PRO-Act» project, particularly workshops with more operational details on specific PRO scales. Additionally, as part of future «PRO-Act» project initiatives, participants will be encouraged to implement pilot projects based on what they learned, which can act as a validation of the contents and structure.

## Supplementary Information

**Additional file 1.** **Table S1.** Feedback questionnaire given to the participants of the workshop.

## Data Availability

Not applicable.

## Abbreviations

ART: Antiretroviral therapy
HCPs: Healthcare professionals
HRQoL: Health-related quality of life
PLHIV: People living with HIV
PROs: Patient reported outcomes
