# Supplementary material for: PRO-Act: a healthcare provider workshop outlining the added value of implementing PROs in routine HIV practice
Source: J Patient Rep Outcomes. 2023 Jun 1;7:50. doi: 10.1186/s41687-023-00584-w (PMC10235267; doi:10.1186/s41687-023-00584-w)
Supplement: Supplementary file 1 — Additional file 1. Table S1. Feedback questionnaire given to the participants of the workshop. [file 41687_2023_584_MOESM1_ESM.docx]

Table S1 – Feedback questionnaire given to the participants of the workshop.

Following your participation in this Workshop, we would like to count on your appreciation, in order to optimize future activities.

​

Thank you in advance for your response to the questions below.

* indicates required field

1. How would you rate the relevance of the content covered in the Workshop?*

- Highly relevant
- Relevant
- Neutral
- Hardly relevant
- Not relevant

1. How likely are you to recommend this Workshop to other Colleagues? *

- Highly likely
- Likely
- Neutral
- Hardly likely
- Not likely

1. What level of impact do you think the content covered in the Workshop will have on your use of PROs (Patient Reported Outcomes) in clinical practice? *

- High impact
- Some impact
- Neutral
- Hardly any impact
- No impact

1. What did you like most about the Workshop?
2. What did you like least about the Workshop?
3. If you wish, leave your suggestions for future initiatives.
